# Clinical findings in 19 cases of invasive pulmonary aspergillosis with liver cirrhosis

**DOI:** 10.1186/2049-6958-9-1

**Published:** 2014-01-08

**Authors:** Jiajia Chen, Qing Yang, Jianrong Huang, Lanjuan Li

**Affiliations:** 1 State Key Laboratory for Diagnosis and Treatment of Infectious Diseases, First Affiliated Hospital, College of Medicine, Zhejiang University, Hangzhou, China; 2 Collaborative Innovation Center for Diagnosis and Treatment of Infectious Diseases, Hangzhou, China

**Keywords:** Antifungal agents, Aspergillosis, Liver cirrhosis, Mortality

## Abstract

**Background:**

*Aspergillus* infection was mostly reported with high mortality rates and a bad prognosis in immunocompromised patients, but data were lacking on the clinical characteristics of aspergillus infection in liver cirrhosis. The aim of this study was to retrospectively assess the morbidity and mortality rate, clinical manifestation, risk factors, and medication of invasive pulmonary aspergillosis (IPA) in liver cirrhosis in The First Affiliated Hospital, College of Medicine, Zhejiang University.

**Methods:**

Patients with liver cirrhosis who had been diagnosed with proven or probable IPA by clinical and laboratory parameters from 1^st^ December 2008 to 1^st^ May2012 were retrospectively evaluated for predisposing factors for IPA and clinical outcome. The follow up ended on 30^th^ July2012. IPA was defined according to European Organization for Research and Treatment of Cancer/Mysoses Study group criteria.

**Results:**

In total, 6,600 patients with liver cirrhosis were enrolled, and 19 out of these developed IPA. Seventeen out of 19 patients died. Imaging findings such as the halo sign and lower respiratory tract infection symptoms contributed to the early diagnosis of IPA. Possible risk factors for IPA included a high Child-Turcotte-Pugh (CTP) score, broad antibiotic usage and steroid exposure. The use of antifungal compounds may prolong a patient’s life.

**Conclusions:**

The mortality of liver cirrhosis with IPA is high. Liver cirrhosis should be considered a risk factor of IPA. Once patients with high CTP scores and steroid and broad spectrum antibiotics exposure present cough and fever, IPA should be taken into consideration and antifungal agents should be used as soon as possible.

## Background

*Aspergillus* species have emerged as an important cause of life-threatening infections in immunocompromised patients with prolonged neutropenia and immunodeficiency who have undergone hematopoietic stem cell transplantation and liver transplantation [1-3]. Moreover, an expanding spectrum of patients at risk for invasive *Aspergillus* has recently been indicated. High risk (allogeneic bone marrow transplant, neutropenia and hematological cancer), intermediate risk (autologous bone marrow transplant, malnutrition, corticosteroids, HIV, solid organ transplant, diabetes, underlying pulmonary diseases and solid organ cancer) and low risk (cystic fibrosis and connective tissue disease) have been described in the literature [4]. Data have shown that invasive aspergillosis is a devastating infectious disease in patients within the intensive care unit even without the presence of an apparent predisposing immunodeficiency [5-7]. Liver cirrhosis has many complications, such as upper gastric bleeding and hepatoencephalopathy, due to portal vein hypertension. All of these complications were fatal. Infection was also a common cause of death. Some studies have focused on invasive pulmonary aspergillosis (IPA) after liver transplantation [3,7]. However, it was rarely reported in patients with liver cirrhosis during pre-transplant period [8].

As such, we retrospectively investigated patients with liver cirrhosis and invasive pulmonary aspergillosis and analyzed their morbility and mortality rate, clinical characteristics and possible risk factors in The First Affiliated Hospital, College of Medicine, Zhejiang University.

## Methods

The study protocol conforms to the ethical guidelines of the 1975 Declaration of Helsinki as reflected in a priori approval by The Human Ethics Committee of the First Affiliated Hospital, School of Medicine, Zhejiang University. Informed consent was obtained from each patient included in the study. The databases of all patients with liver cirrhosis admitted to The First Affiliated Hospital, College of Medicine, Zhejiang University from 1^st^ December 2008 to 1^st^ May 2012 were reviewed. The follow up continued until the date of death, the date of last hospital visit, or the end of the study (30^th^ July 2012). The minimum follow up period for enrolled patients was 3 months after discharge. Medical and microbiological records were reviewed. Patients’ demographics, duration of admission, predisposing factors, clinical features, treatments and outcomes were noted.

Patients with HIV negative conforming to the definition of IPA were included. IPA was defined according to the European Organization for Research and Treatment of Cancer/Invasive Fungal Infections Cooperative Group and the National Institute of Allergy and Infectious Diseases Mycoses Study Group criteria [9]. The following factors contributed to the diagnosis of probable IPA: positive culture of *Aspergillus spp.* from sputum specimens; bilateral nodular infiltrates with central cavitation, bilateral alveolar infiltrates or homogeneous infiltrates of the right upper lobe; halo sign or the air-crescent sign (a macronodule surrounded by a ground-glass opaque perimeter) on a computed tomography (CT) scan; at least two minor clinical findings (signs of lower respiratory tract infection, pleural rub or the presence of any new infiltrate in a patient who did not fulfill the major criteria but for whom no alternative diagnosis was available). A definite diagnosis requires the presence of fungi in tissue by biopsy or needle aspiration. In brief, the appearance of pulmonary consolidation or infiltrate and rapid progression on thoracic imaging with antibiotic-resistant fever in the appropriate host setting and positive sputum culture was diagnosed as probable IPA. With tissue evidence, the patient was diagnosed with proven IPA.

The following laboratory parameters were also used to diagnose IPA: white blood cell count, differential leucocyte count, albumin, globulin, total bilirubin, international normalized ratio (INR), creatinine and C-reactive protein (CRP), all of which were measured using standard techniques.

SPSS version 16.0 software was used for the statistical analyses. Continuous variables were summarized as either mean ± SD or medians with interquartile ranges. For categorical variables, the percentages of patients were calculated. Continuous variables were compared by t-test, and p < 0.05 was considered statistically significant for all analyses.

## Results

Between December 2008 and May 2012, a total of 6,600 patients were diagnosed with liver cirrhosis and admitted to the State Key Laboratory for Diagnosis and Treatment of Infectious Diseases, Department of Infectious Diseases, The First Affiliated Hospital, College of Medicine, Zhejiang University, China.

Out of these patients, 19 were also diagnosed with probable or definite IPA according to the diagnostic criteria. The morbility of IPA in liver cirrhosis was 0.288%. The demographic and clinical characteristics of the 19 patients with IPA and liver cirrhosis are presented in Table 1.

**Table 1:** Demographic and clinical characteristics of patients with liver cirrhosis diagnosed with invasive pulmonary aspergillosis (n = 19)

**Characteristic**	**Patients (n= 19)(%)**
Sex (males/females)	12/7
Age, years, median (range)	54 (30–75)
Underlying liver disease	
Hepatitis B virus	11 (57.89)
Alcoholic	3 (15.79)
Autoimmune hepatitis	1 (5.26)
Hybrid cause	4 (21.05)
Basic underlying disease	
Cardiovascular disease	4 (21.05)
Diabetes mellitus type 2	2 (10.53)
Chronic lung diseases	2 (10.53)
Complications	
Hepatic encephalopathy	8 (42.10)
Hepatic renal syndrome	4 (21.05)
Upper gastric bleeding	4 (21.05)
Ascites	16 (84.21)
Hydrothorax	10 (52.63)
Hepatic carcinoma	2 (10.53)
CTP scores (mean ± SD)	11 ± 2
Child B	6 (31.58)
Child C	13 (68.42)
MELD (mean ± SD)	22 ± 7
Neutropenia	2 (10.53)
Broad-spectrum antibiotics usage	18 (94.74)
One antibiotic	9 (47.37)
Two antibiotics	4 (21.05)
Three antibiotics	5 (26.32)
Steroids exposure(longer than 1 week)	11 (57.89)
Survival rate	
1 month	5/19
3 month	4/19

Data presented as *n* patients or mean ± SD unless stated otherwise.

CTP, Child-Turcotte-Pugh; MELD, Model for End-Stage Liver Disease.

The subjects consisted of 12 men and 7 women with a median age of 54 years (range, 30–75 years). Most of the patients with liver cirrhosis and IPA had one or several complications such as hepatic encephalopathy, ascites, hemorrhage, hepatorenal syndrome or hepatic carcinoma. Only two patients had neutropenia. Child-Turcotte-Pugh (CTP) scores of all patients were Child B or C. The mean Model for End-Stage Liver Disease score was 22 ± 7. Most patients received broad-spectrum antibiotics and steroids. Despite the antifungal therapy, most (9/19) patients were dead within 7 days. Only 2 patients survived without lung infections before the last investigation.

All patients with IPA had mild to moderate fever (38–39°C), cough, chest pain and crackles, while 9 patients had hemoptysis; these were not specific symptoms but may have been useful indicators of early stage IPA (Table 2). A total of 16 patients presented with at least one macronodule on chest CT, whose regional distribution in the lungs was as expected (Table 2). The remaining 8 patients had at least one halo or air-crescent sign. All patients were treated with antifungal therapy as soon as the culture result was positive.

**Table 2:** Clinical and imaging findings and antifungal therapy of patients with liver cirrhosis and IPA (n = 19)

**Finding**	**Patients (n=19) (%)**	
Clinical characteristics		
Fever	19 (100)	
Cough	19 (100)	
Chest pain	19 (100)	
Hemoptysis	9 (47.37)	
Crackles	19 (100)	
Imaging findings (X-ray or CT)		
Changes in bilateral lung fields	16 (84.21)	
Bilateral lung fields diffused	16 (84.21)	
Right unilateral lung	3 (15.7)	
Halo or air-crescent sign	8 (42.10)	
Laboratory parameters	Pre-diagnosis	Post-diagnosis
CRP, mg/dL	-	34.0 ± 21.1
Leukocyte counts, × 10^9^/L	9.3 ± 5.5	11.3 ± 6.5
Neutrophil, %	74.0 ± 15.0	81.3 ± 9.3

Data presented as *n* patients or mean ± SD unless stated otherwise.

CT, computed tomography; CRP, C-reactive protein.

Six patients were treated with caspofungin, whereas six with voriconazole, one with itraconazole, and one with amphotericin B. Five patients died before the culture results were available and had not used any anti-*Aspergillus* agents. Only 2 patients treated with voriconazole were still alive before the last investigation. The survival time of the 17 patients from the diagnosis of IPA was 1–162 days. Those using itraconazole or amphotericin B all died within 7 days. Most (4/6) of the patients taking caspofungin died within 7 days, while only one survived for 102 days. The all-causes mortality rate of liver cirrhosis patients with IPA was 89.47% (17 of 19 patients). IPA attributable mortality was 57.89% (11/19). The rate of haemorrhage**-**caused mortality was 10.53 % (2/19), while that of hepatorenal syndrome (HRS)**-**attributable mortality was 21.05% (4/19).

## Discussion

IPA is an opportunistic infection in immunocompromised or immunodeficient patients. As making the early diagnosis of invasive *Aspergillus* infections is difficult, numerous factors have been studied to identify patients at high risk in the hope that antifungal prophylaxis and empirical treatment will improve survival. The 2004 consensus defined cancer, whether treated or not, and hematopoietic stem cell transplantation as host risk factors [9]. The 2009 consensus included immunocompromised patients [10] as a new host risk factor, but did not include critically ill patients. IPA was mostly reported in cancer, hematopoietic stem cell transplantation and liver transplantation. It has also recently been described in critically ill patients, even those without risk factors [7].

Although data have shown that invasive *Aspergillus* may occur in liver transplantation [1,3,11], it has rarely been reported in other liver diseases. Much uncertainty remains in liver cirrhosis. In our study, about 3 per 1,000 patients with liver cirrhosis had IPA. All patients had high CTP scores. It was suggested that severe decompensated liver cirrhosis may be a host risk factor for the development of invasive aspergillosis. Other risk factors like neutropenia, diabetes and underlying pulmonary diseases were present in part of the group. Most of those patients had used antibiotics and steroids, which could have prevented possible bacterial infections and reduced inflammation. However, these treatments were harmful to the host’s immune function and did not cover all possible pathogens; therefore, patients were prone to many types of infections caused by opportunistic pathogens including *Aspergillus* spp. Cell-mediated immunity is the basis of fungal resistance. The respiratory epithelium was usually damaged by the invasive operation and medication in the hospital. Then insufficient secretion of cytokines and chemokines by the macrophages also plays an important role in the coordination of innate and adaptive cellular responses to aspergillus infection [12]. T-lymphocyte helper (Th) cells exhibited successful selection and clonal expansion of direct professional effector cells against *Aspergillus*[13,14]. However, as mentioned in the literature, the lymphocyte counts were very low and the T-lymphocyte counts were inversely related to the severity of cirrhosis [15]. Reduced cellular immune function might contribute to the increased infectious morbidity of these patients with liver cirrhosis.

Both culture results and imaging findings were important for IPA diagnosis. Culture results may take longer than imaging findings. Imaging findings, such as a halo or air-crescent sign, are useful for the diagnosis of IPA and could guide antifungal therapy at an early stage [16,17]. In our study, all patients underwent CT or X-ray imaging as well as sputum culturing. The galactomannan (GM) test was not routinely performed to discover *Aspergillus* infections earlier. As far as laboratory parameters were concerned, no specific biomarkers for IPA were available. Patients with liver cirrhosis and IPA did not have significantly increased neutrophil numbers (*p = 0.253)* or proportions *(p = 0.137)*; however, they had higher than normal CRP levels, which may be a clue for the diagnosis of IPA and liver cirrhosis while combined with imaging findings.

The mortality rates of *Aspergillus* infection have been reported to be 40–90% in high-risk populations and depend on host factors, infection site and treatment regimen applied [18]. In our study, approximately 20% of patients were alive after the 3-month investigation, but most died shortly after diagnosis. Multiple factors determined the survival rates. Severe cirrhosis and pulmonary infections both induced high mortality. Invasive aspergillosis may increase the risk of death. Two patients were alive in the last investigation. Organs including the lung, brain and eyeballs were involved in proven cases. Although one patient’s CTP score was C, he was young enough to be cured by an antifungal agent and did not have other complications.

We know that voriconazole may induce liver and kidney injuries. The dose should be adjusted according to underlying kidney and liver diseases. In the two surviving patients, we did not change the dose even with abnormally high total bilirubin levels, and despite the poor prognosis of aspergillosis, liver and renal function did not worsen after treatment and they still recovered progressively. The caspofungin dose did not need to be changed and the patient survival time was prolonged to up to 102 days.

## Conclusions

In conclusion, patients with advanced or decompensated liver cirrhosis were immunocompromised hosts who were prone to IPA. Broad-spectrum antibiotics and steroid usage may be predisposing factors. When a GM test cannot be performed and symptoms of pneumonia such as cough or hemoptysis in liver cirrhosis appear, IPA should be considered. If a GM test can be performed as early as possible, a patient’s prognosis may be improved. Since gathering tissue evidence can be difficult, early pre-emptive treatment with antifungals is warranted. Triazoles like voriconazole, itraconazole and echinocandin as well as caspofungin can improve patient prognosis. Although the use of voriconazole without adjusting the dose did not impact negatively liver and renal function in the current study, we should closely monitor liver and renal function effects in patients with liver cirrhosis.

### Availability of supporting data

The data set supporting the results of this article is included within the article.

## Abbreviations

CRP: C-reactive protein; CT: Computed tomography; CTP: Child-Turcotte-Pugh; HIV: Human immunodeficiency virus; GM: Galactomannan; INR: International normalized ratio; IPA: Invasive pulmonary aspergillosis; HRS: Hepatorenal syndrome; MELD: Model for end-stage liver disease; Th: T-lymphocyte helper.

## Competing interests

The authors declare that they have no competing interests.

## Authors’ contributions

JC, QY and LL participated in the design of the study, analysis and interpretation of data, and drafting of the manuscript. JH participated in the interpretation of data and critical review of the manuscript for important intellectual content. All authors read and approved the final manuscript.
